# A Comparative Study on ZrO_2_- and MgO-Based Sulfonic Acid Materials for the Reactive Adsorption of *o*-Xylene

**DOI:** 10.3390/molecules30153171

**Published:** 2025-07-29

**Authors:** Hongmei Wang, Xiaoxu Zhang, Ziqi Shen, Zichuan Ma

**Affiliations:** 1 Hebei Key Laboratory of Inorganic Nano-Materials, College of Chemistry and Material Science, Hebei Normal University, Shijiazhuang 050024, China; wanghm@stu.hebtu.edu.cn (H.W.); shenziqi@stu.hebtu.edu.cn (Z.S.); 2 School of Environmental Science and Engineering, Hebei University of Science and Technology, Shijiazhuang 050018, China

**Keywords:** *o*-xylene, chlorosulfonic acid modification, ZrO_2_, MgO, adsorption

## Abstract

The recovery and abatement of volatile organic compounds (VOCs) have received increasing attention due to their significant environmental and health impacts. Supported sulfonic acid materials have shown great potential in converting aromatic VOCs into their non-volatile derivatives through reactive adsorption. However, the anchoring state of sulfonic acid groups, which is closely related to the properties of the support, greatly affects their performance. In this study, two supported sulfonic acid materials, SZO and SMO, were prepared by treating ZrO_2_ and MgO with chlorosulfonic acid, respectively, to investigate the influence of the support properties on the anchoring state of sulfonic acid groups and their reactive adsorption performance for *o*-xylene. The supports, adsorbents, and adsorption products were extensively characterized, and the reactivity of SZO and SMO towards *o*-xylene was systematically compared. The results showed that sulfonic acid groups are anchored on the ZrO_2_ surface through covalent bonding, forming positively charged sulfonic acid sites ([O_1.5_Zr-O]^δ−^-SO3H^δ+^) with a loading of 3.6 mmol/g. As a result, SZO exhibited excellent removal efficiency (≥91.3%) and high breakthrough adsorption capacity (ranging from 38.59 to 82.07 mg/g) for *o*-xylene in the temperature range of 130 –150 °C. In contrast, sulfonic acid groups are anchored on the MgO surface via ion-paired bonding, leading to the formation of negatively charged sulfonic acid sites ([O_0.5_Mg]^+^:OSO_3_H^−^), which prevents their participation in the electrophilic sulfonation reaction with *o*-xylene molecules. This work provides new insights into tuning and enhancing the performance of supported sulfonic acid materials for the resource-oriented treatment of aromatic VOCs.

## 1. Introduction

Volatile organic compounds (VOCs) are a category of organic compounds that exhibit volatility under normal temperature conditions and possess high vapor pressures. These compounds are ubiquitously present in various emission sources, including industrial processes, transportation activities, and daily human activities. Representative VOCs include benzene, toluene, xylene, formaldehyde, ethanol, alkanes, and alkenes, among others [1,2,3,4]. Despite their relatively low atmospheric concentrations, VOCs have garnered significant global attention due to their high reactivity, diverse chemical compositions, and extensive emission sources. VOCs not only serve as precursors to photochemical smog and ozone pollution, thereby severely impacting air quality, but also exhibit notable toxicity and carcinogenic properties, posing potential risks to human health [5,6,7]. For instance, benzene compounds can adversely affect the central nervous system. Furthermore, certain highly toxic VOCs have been classified as carcinogens by the World Health Organization (WHO). Additionally, the persistent release of VOCs contributes to indoor air pollution, soil contamination, groundwater degradation, and ecological system instability [8,9,10,11]. In light of the environmental and health hazards associated with VOCs, the advancement of efficient and eco-friendly VOC recovery and emission reduction technologies has emerged as a critical research focus within the realm of contemporary environmental governance [12,13].

The development of reduction and recovery technologies for VOCs has been diverse, encompassing methods such as extraction, condensation, membrane separation, photocatalytic decomposition, thermal catalytic oxidation, and microbial degradation, among others [14,15,16,17,18,19,20,21,22,23]. Among these approaches, the adsorption method has emerged as the preferred technical solution for VOC pollution control, due to its advantages of high efficiency, low operating costs, simplicity of operation, safety and reliability, and the recyclability of the adsorbent [24]. In industrial applications, the regeneration of adsorption devices is primarily achieved through two processes: temperature swing adsorption (TSA) and pressure swing adsorption (PSA) [25,26,27]. While TSA technology enables thorough regeneration and is particularly effective for the removal of trace or difficult-to-desorb impurities, it also presents challenges such as high energy consumption, significant equipment investment, and extended operating cycles. In contrast, the PSA process offers a shorter cycle period, higher adsorbent utilization rates, and good product purity; however, its recovery rate remains relatively low. In general, the structural characteristics of adsorbents, including the pore volume, specific surface area, and pore size distribution, serve as the critical determinants of adsorption performance [28,29,30]. Moreover, the adsorbent materials widely used at present (such as activated carbon and zeolite) also face several challenges in practical applications. For example, they are prone to pore blockage, have a limited service life, and have high regeneration costs. These technical problems have somewhat restricted their large-scale application in the industrial field [31,32]. Consequently, the development of novel high-efficiency adsorbent materials and the optimization of the regeneration process are crucial for enhancing the technological level of VOCs’ treatment.

In our prior research, we innovatively developed a novel reactive adsorption technology featuring sulfonic acid functional groups for the simultaneous treatment and resource recovery of aromatic organic pollutants [33]. The technology involves preparing a composite adsorbent (SSSA) by modifying silica gel with sulfuric acid, enabling the efficient capture of *o*-xylene gases through a directional sulfonation reaction. Unlike conventional PSA and TSA processes, this method offers three distinct advantages: in situ removal of aromatic pollutants, controllable recovery of aromatic sulfonic acids as products, and regeneration/recycling of the carrier material. In-depth investigations revealed that the SSSA surface contains two types of sulfonic acid groups: P-state groups physically loaded onto the surface and C-state groups chemically bonded to the carrier [33,34,35]. The C-state sulfonic acid groups exhibited superior removal efficiency for *o*-xylene due to their stable chemical bonding structure with the silica gel carrier. Furthermore, adjusting the composition or structure of the carrier, optimizing the material preparation process, and modulating the reaction environment can effectively regulate the proportion of C-state sulfonic acid groups within the SSSA material. By precisely controlling the distribution density of hydroxyl groups on the silica gel carrier surface, the proportion of Si-O-S bonded sulfonic acid groups (a specific type of bridging oxygen-linked sulfonic acid groups) can be finely tuned. When silicon atoms are substituted with hetero-elements, the electronic structure of sulfur undergoes significant changes due to the electron reconstruction effect induced by the substituting elements, potentially altering the coordination environment and reactivity of the bridging oxygen-linked sulfonic acid groups. Therefore, systematically exploring the structure–activity relationship of these bridging oxygen-linked sulfonic acid groups is critical for optimizing the performance of functionalized materials.

**Scheme 1:**
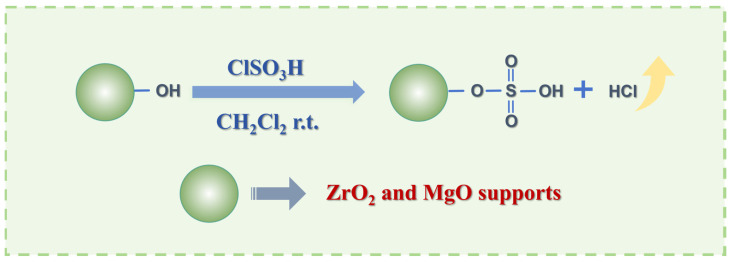
Preparation process for the SZO and SMO.

Based on the aforementioned considerations, we systematically constructed a comparative research framework for the first time. Zirconium dioxide (ZrO_2_, abbreviated as ZO) and magnesium oxide (MgO, abbreviated as MO) were selected as alternative materials to replace traditional silica supports. The focus of the research was on precisely analyzing C-state sulfonic acid groups on the surfaces of different supports and thoroughly exploring the reaction–adsorption synergistic mechanism during the removal process of *o*-xylene. Given the substantial differences in the electronic structures of ZO and MO, commercial ZO and MO powders with a similar particle size, specific surface area, and pore volume were chosen as experimental supports to ensure consistent experimental conditions. Through this design, the influence of oxide support properties on the characteristics of generated sulfonic acid groups could be elucidated more clearly. Additionally, ZO and MO supports were modified with chlorosulfonic acid in a dichloromethane medium to selectively generate C-state sulfonic acids (denoted as SZO and SMO, respectively), and the detailed steps are illustrated in Scheme 1 [36]. The research findings revealed that the loading amount of sulfonic acid groups in SMO was significantly higher than that in SZO. However, regarding the reaction–adsorption performance of *o*-xylene, SZO demonstrated remarkable superiority. Under specific experimental conditions, the breakthrough reaction–adsorption capacity of SZO reached 82.07 mg/g, whereas the reaction–adsorption capacity of SMO was nearly negligible. This phenomenon suggests that the number of sulfonic acid groups is not the sole determinant of reaction–adsorption performance, and the electronic configuration of sulfonic acid groups may play a more critical role. This significant difference can be attributed to the distinct electrical properties of sulfonic acid groups in the two functional materials. In summary, this study provides a robust theoretical foundation and experimental evidence for the development of high-performance supported sulfonic acid materials and their application in the resource recovery of VOCs.

## 2. Results and Discussion

### 2.1. Analysis of the Basic Properties of ZO and MO Carrier Materials

**Figure 1:**
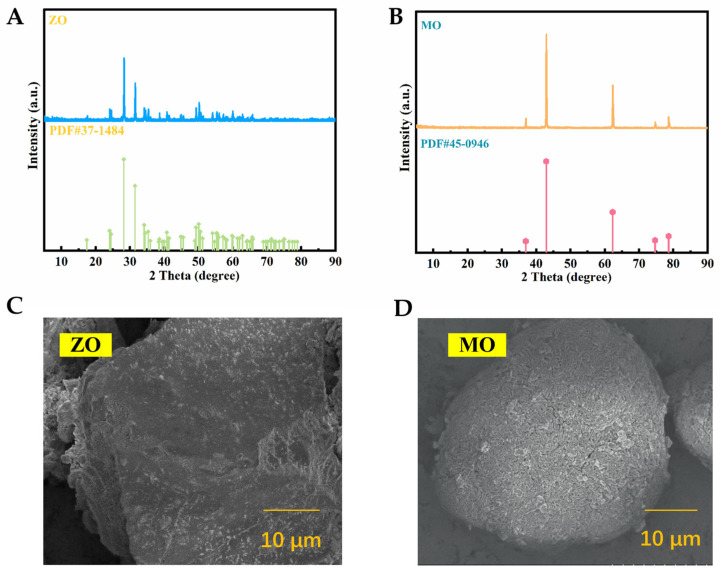
(**A**) XRD pattern of ZO; (**B**) XRD pattern of MO; (**C**) SEM image of ZO; (**D**) SEM image of MO.

As this study focuses on fundamental research, commercially available reagent-grade ZO and MO powders were directly selected as carrier materials and subsequently modified using chlorosulfonic acid. Figure 1A,B show the X-ray diffraction (XRD) patterns of the selected carriers, ZO and MO, respectively, confirming their crystalline structures. Despite some background noise, the sharp diffraction peaks of ZO (Figure 1A) align well with the standard diffraction card (PDF#37-1484), indicating a monoclinic crystal structure with lattice parameters a = 5.313 Å, b = 5.213 Å, and c = 5.147 Å, corresponding to the P21/a space group [37]. MO exhibits characteristic peaks at 2θ values of 36.93°, 42.91°, 62.30°, 74.68°, and 78.62° (Figure 1B), which match the standard pattern (PDF#45-0946), verifying its cubic crystal structure (a = b = c = 4.221 Å, space group Fm-3m) [38]. Scanning electron microscopy (SEM) images of ZO and MO are shown in Figure 1C,D, respectively. ZO exhibits a relatively smooth morphology with localized nanoscale roughness, whereas MO crystals present as agglomerated blocks consisting of relatively smooth micrometer-scale surfaces.

**Figure 2:**
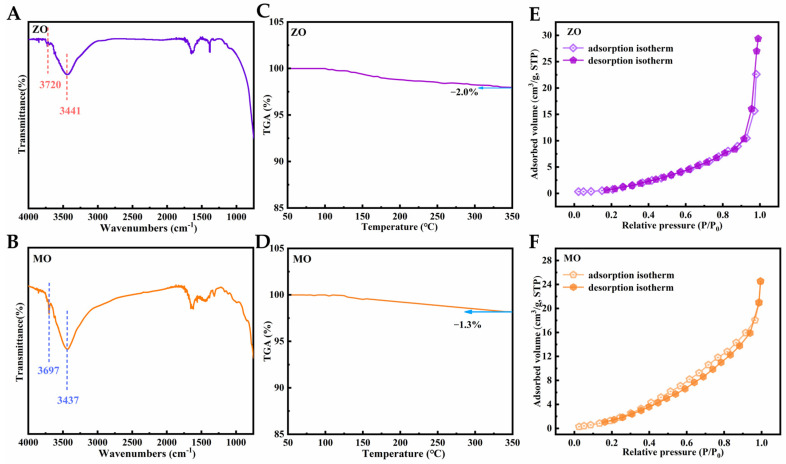
(**A**) FTIR spectra of ZO; (**B**) FTIR spectra of MO; (**C**) TG curves of ZO; (**D**) TG curves of MO; (**E**) N_2_ adsorption–desorption isotherm of ZO; (**F**) N_2_ adsorption–desorption isotherm of MO.

As shown in the FTIR spectra (Figure 2A,B), both materials exhibit characteristic hydroxyl vibrations: broad peaks at 3441 cm^−1^ and 3437 cm^−1^ (attributed to hydrogen-bonded hydroxyl groups or adsorbed water molecules), along with well-defined sharp bands at 3720 cm^−1^ and 3697 cm^−1^ (characteristic of non-hydrogen-bonded hydroxyl groups). The presence of these higher-frequency peaks confirms that the surfaces of ZO and MO possess chemically active hydroxyl groups capable of participating in catalytic reactions [39,40]. The thermogravimetric analysis (TGA) results (Figure 2C,D) show that, during heating from 50 °C to 350 °C at a rate of 5 °C/min, the weight losses of ZO and MO were only 2.0% and 1.3%, respectively, demonstrating their extremely low hygroscopicity and high thermal stability. Collectively, these properties render ZO and MO as ideal carriers for sulfonic acid group immobilization. Figure 2E,F display the N_2_ adsorption–desorption isotherms of ZO and MO, respectively. Both isotherms conform to the Type III pattern as classified by the International Union of Pure and Applied Chemistry (IUPAC) and show no significant hysteresis loop between the adsorption and desorption branches [41,42]. Based on BET analysis, the specific surface areas of ZO and MO were calculated to be 9.53 m^2^/g and 15.32 m^2^/g, respectively, demonstrating a similar range. This similarity offers a reliable foundation for investigating how variations in their intrinsic properties affect their sulfonic acid functionalization behavior.

### 2.2. Comparison of Sulfonic Acid Loading in SZO and SMO Materials

**Figure 3:**
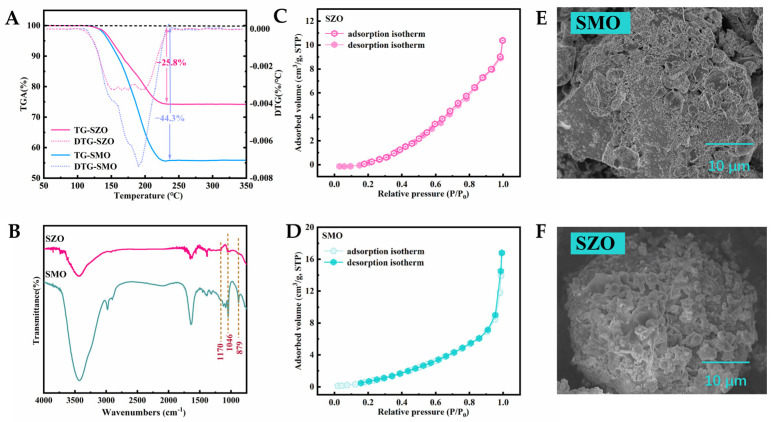
(**A**) TG curves and DTG curves of SZO and SMO; (**B**) SEM images of SZO; (**C**) SEM images of SMO; (**D**) N_2_ adsorption–desorption isotherms of SZO; (**E**) N_2_ adsorption–desorption isotherms of SMO; (**F**) FTIR spectra of SZO and SMO.

Following the method described in Section 3.2, the surfaces of ZO and MO were chemically modified using chlorosulfonic acid. Figure S1A,B show the X-ray diffraction (XRD) patterns of SZO and SMO, respectively. For SZO, the primary diffraction peaks aligned well with the standard reference pattern (PDF#37-1484) [37], indicating that its structure remained stable. Regarding SMO, most diffraction peaks corresponded to the reference pattern PDF#45-0946 [38], suggesting that the material predominantly adopted an MO framework structure. However, a few additional peaks were observed, which may be attributed to the presence of minor magnesium sulfonate impurities. The sulfonic acid group loadings of SZO and SMO were quantified by acid–base back titration and determined to be 3.6 ± 0.1 mmol/g and 5.5 ± 0.1 mmol/g, respectively [34,35], indicating that SZO possessed approximately 0.65 times the sulfonic acid content of SMO. The thermogravimetric analysis (TGA) and derivative thermogravimetric (DTG) curves (Figure 3A) revealed significant mass losses for SZO and SMO in the temperature range of 120–250 °C, with values of 25.8% and 44.3%, respectively. These losses are primarily attributed to the thermal decomposition of surface-bound sulfonic acid groups, and the weight loss ratio is consistent with the titration results. Furthermore, the NH_3_-TPD peak area calculations (Figure S2) showed a SZO/SMO sulfonic acid group density ratio of 0.6, further corroborating the higher sulfonic acid loading in SMO. As illustrated in Scheme 1, this distinction can be attributed to differences in surface hydroxyl density [43], suggesting that MO contains a higher concentration of surface hydroxyls than ZO. SEM images (Figure 3B,C) illustrated the morphological evolution of SZO and SMO after modification. Compared to unmodified ZO and MO (Figure 1C,D), the treated materials exhibited rougher surfaces and the formation of visible pores. The EDX results (Figure S3) confirmed that S elements were detected on the surfaces of both modified SZO and SMO. At the same time, the relative content of O elements significantly increased. These changes can be attributed to the etching effect of chlorosulfuric acid and the successful anchoring of sulfonic acid groups [44,45]. The BET analysis of N_2_ adsorption–desorption isotherms (Figure 3D,E) showed decreases in the specific surface area to 3.55 m^2^/g for SZO and 8.16 m^2^/g for SMO, representing reductions of 5.98 m^2^/g and 7.16 m^2^/g, respectively. These findings confirm a negative correlation between the sulfonic acid loading and specific surface area. It is worth noting that the pore size distribution analysis (Figure S4) shows that the pore size increases after sulfonation modification, which can be attributed to the partial etching effect of ClSO_3_H on the metal oxide framework [36]. FTIR spectroscopy (Figure 3F) further validated the surface modification, with clear absorption bands at 879 cm^−1^ (S–O stretching), 1046 cm^−1^ (S=O symmetric stretching), and 1170 cm^−1^ (S=O asymmetric stretching), characteristic of sulfonic acid groups [44,45]. XPS survey analysis (Figure S5A,B) further verified the chemical composition of the modified materials: the coexistence of Zr, S, and O elements was detected in SZO, while the original ZO only contained Zr and O; similarly, Mg, S, and O elements were present in SMO, while MO only contained Mg and O. In addition, the analysis of the high-resolution S 2p spectra (Figure S5C,D) showed that the binding energy doublets of S 2p3/2 (168.69 eV) and S 2p1/2 (169.84 eV) in SZO, as well as the doublets of S 2p3/2 (168.26 eV) and S 2p1/2 (169.44 eV) in SMO, were both consistent with the typical binding energy range of the sulfonic acid group (–SO_3_H), further confirming the stable anchoring of the sulfonic acid group on the material surface. Overall, the combined SEM, BET, and FTIR characterization results clearly confirm the successful immobilization of sulfonic acid groups on the surfaces of SZO and SMO.

### 2.3. Comparative Analysis of o-Xylene Removal Reactivity by SZO and SMO

**Figure 4:**
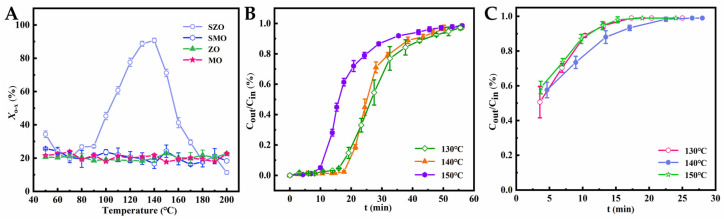
(**A**) Relationship curves between *X*_o-x_ and *T*; (**B**) reactive adsorption breakthrough curves in SZO at different temperatures; (**C**) reactive adsorption breakthrough curves in SMO at different temperatures.

As shown in Figure 4A, the reaction temperature was gradually increased to investigate the effects of SZO and SMO, along with their unmodified supports (ZO and MO), on the removal efficiency of *o*-xylene (*X*_o-x_). The removal rate–temperature profiles enabled evaluation of the reactivity of sulfonic acid-functionalized materials toward aromatic VOCs [33,34,35]. The experimental results revealed that the *o*-xylene removal efficiencies of unmodified ZO and MO remained relatively stable between 15.6% and 28.2%, consistent with their limited physical adsorption capacity. In contrast, SZO exhibited a marked temperature-dependent pattern, with its *o*-xylene removal efficiency displaying an inverted “U”-shaped trend. Within the reactive adsorption temperature range of 130–150 °C, SZO achieved removal efficiencies above 91.3%, demonstrating superior reactivity. Conversely, SMO showed limited activity, with removal rates consistently below 26.3%, resembling the behavior of unmodified MO and indicating a lack of significant chemical interaction with *o*-xylene. To further evaluate and compare the reactive adsorption capacities of SZO and SMO, an isothermal breakthrough curve analysis was conducted. This method quantitatively described the adsorption dynamics under specific conditions. Breakthrough curves for SZO and SMO (Figure 4B,C), along with the key parameters *t*_B_ and *Q*_B_ presented in Table 1, depict clear performance differences. For SZO, both *t*_B_ and *Q*_B_ increased with temperature, ranging from 9.65 min and 38.59 mg/g at 130 °C to mg/g at 150 °C, respectively. These results suggest that 140 °C is the optimal temperature for efficient reactive adsorption, consistent with trends observed in other SiO_2_-based materials, although those exhibit relatively lower performance, potentially due to lower sulfonic acid loadings [34]. In contrast, SMO exhibited negligible reactivity under identical conditions. Upon pollutant introduction, the adsorption bed broke through almost immediately, resulting in minimal *t*_B_ and *Q*_B_ values. This stark difference cannot be fully attributed to the lower sulfonic acid loading of SMO. While group density plays a role, other factors must contribute to the observed performance gap. These observations highlight the need for further investigation into the mechanistic differences governing SZO and SMO behavior in *o*-xylene removal.

### 2.4. Investigation into the Fixation State of Sulfonic Acid Groups and Their Operational Mechanism in o-Xylene Elimination Procedures

**Figure 5:**
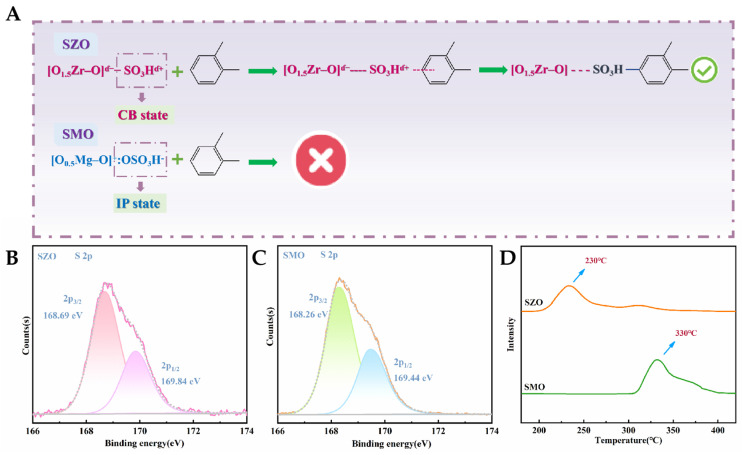
(**A**) Reactive adsorption equations of the CB-state sulfonic acid in SZO and the IP-state sulfonic acid in SMO with *o*-xylene; (**B**) XPS spectrum of S 2p of SZO; (**C**) XPS spectrum of S 2p of SMO; (**D**) NH_3_-TPD curves of SZO and SMO.

The experimental data reveal a significant difference in sulfonic acid group loading between SZO (3.6 mmol/g) and SMO (5.5 mmol/g), which can be attributed to the distinct hydroxyl group densities on the carrier surfaces. Specifically, the density of active hydroxyl sites per unit area on the MO surface is significantly higher than that on ZO, enabling a higher degree of functional group grafting during sulfonation. Assuming a uniform distribution and identical chemical properties of sulfonic acid groups in both materials, SMO would theoretically be expected to exhibit superior catalytic activity and adsorption capacity. However, the deviation between the experimental results and theoretical predictions suggests that the actual chemical state of the sulfonic acid groups may be regulated by carrier characteristics, with essential differences in their anchoring patterns. This finding aligns with our previously proposed dual-state anchoring theory for sulfonic acid groups: covalent bond (CB) and ionic pair (IP) bonding modes significantly influence the apparent performance of functional groups. In the SiO_2_ system, the small electronegativity difference between Si (χ = 1.90) and O (χ = 3.44) enables CB-state sulfonic acid groups to exhibit excellent *o*-xylene adsorption performance through strong covalent interactions. Although there is a certain electronegativity difference between Zr (χ = 1.33) and O (χ = 3.44), the high charge density of Zr^4+^ induces a strong polarization effect on O, resulting in a more similar charge distribution in the Zr-O bond and facilitating the formation of CB-state sulfonic acid groups during chlorosulfonic acid modification [46]. As shown in Figure 5A, sulfonic acid sites in SZO are positively charged in the CB state, enabling electrophilic substitution reactions with *o*-xylene molecules, particularly in sulfonation [47,48]. However, due to the slightly larger electronegativity difference (Δ = 2.11) between Zr-O and Si-O (Δ = 1.54), the adsorption performance of SZO remains lower than that of SiO_2_-based materials. For the MgO system, the electronegativity difference (Δ = 2.13) between Mg (χ = 1.31) and O (χ = 3.44) triggers significant electron transfer, promoting sulfonic acid groups to anchor in the IP state. This interaction converts sulfonic acid groups into negatively charged sulfate hydrogen groups ([O_0.5_Mg]^+^:OSO_3_H^−^), inhibiting their participation in electrophilic sulfonation reactions with *o*-xylene molecules.

Further verification of the differences in sulfonic acid groups between SZO and SMO in the CB and IP states was conducted using XPS and NH_3_-TPD characterization. As depicted in Figure 5B,C, the S 2p core-level spectra of SZO relative to SMO are presented. Subsequent Gaussian fitting decomposition of the peaks revealed two characteristic peaks corresponding to the S 2p3/2 and S 2p1/2 orbitals [49]. The experimental results indicate that the binding energies of S 2p3/2 (169.84 eV) and S 2p1/2 (168.69 eV) in SZO are systematically positive-shifted compared to those in SMO (169.44 eV and 168.26 eV). This systematic change in binding energy suggests that sulfur atoms in CB-state sulfonic acid groups possess significantly higher positive charge density (δ^+^) than those in the IP state. These findings further support the hypothesis of distinct anchoring states of sulfonic acid groups in SZO and SMO. As shown in Figure 5D, within the temperature range of 50 –450 °C, NH_3_-TPD analysis revealed single ammonia desorption peaks for SZO and SMO, with notable differences in the center positions, intensities, and areas. Specifically, the highest desorption temperature for SMO was 330 °C, whereas it was only 230 °C for SZO. Based on the experimental process, it can be inferred that the hydrogen bond strength between NH_3_ molecules and sulfonic acid groups on the material surface is stronger for IP-state sulfonic acid groups than for CB-state sulfonic acid groups. Thus, the lower desorption peak temperature of SZO aligns with the characteristics of CB-state sulfonic acid groups, indicating the weaker binding of NH_3_ molecules to SZO compared to SMO. This phenomenon correlates with the chemical characteristics of sulfonic acid groups in the two materials: sulfonic acid groups in SMO predominantly exist in the IP state, while those in SZO mainly reside in the CB state [50]. Quantitative analysis of the NH_3_-TPD peak areas further revealed that the ammonia desorption peak area of SZO is 0.6 times that of SMO, reflecting the relative density of anchored sulfonic acid groups, which was consistent with the measured loading amounts.

**Figure 6:**
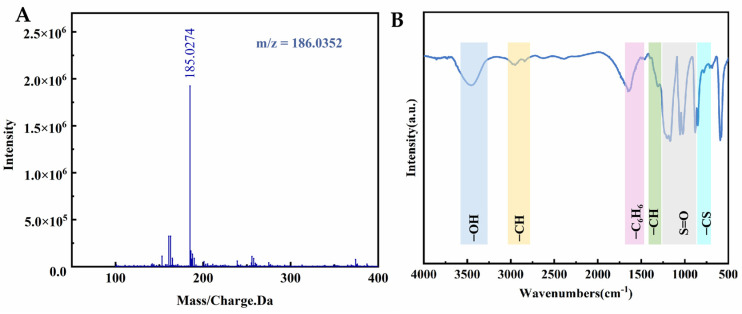
(**A**) ESI-MS spectrum of the adsorption product; (**B**) FTIR spectrum of the adsorption product.

In conclusion, the CB state of sulfonic acid groups in SZO exhibits significant sulfonation reaction activity, greatly enhancing its reactive adsorption capacity for *o*-xylene. This finding corroborates previous research on sulfonic acid-functionalized silica for *o*-xylene removal. It is reasonable to speculate that the primary adsorption product formed during the reaction between SZO and *o*-xylene is 3,4-dimethylbenzenesulfonic acid (as shown in Figure 5A). To verify this assumption, methanol was used as an extraction solvent, and comprehensive analysis of reaction products was performed using high-performance liquid chromatography–mass spectrometry (HPLC-MS) and Fourier transform infrared spectroscopy (FTIR). Researchers obtained the mass spectrum via HPLC-MS in negative ion mode. As shown in Figure 6A, a prominent molecular ion peak was observed at m/z = 185, corresponding to a relative molecular mass of 186, which matches the theoretical prediction [50]. This result strongly supports the existence of 3,4-dimethylbenzenesulfonic acid as the adsorption product.

Furthermore, FTIR characterization confirmed the chemical structure of the product. Using the liquid film method, the obtained infrared spectrum exhibited a series of characteristic absorption peaks (see Figure 6B): a broad peak at 3441 cm^−1^ corresponds to O-H stretching vibrations; peaks at 2920 and 2838 cm^−1^ reflect asymmetric and symmetric C-H stretching vibrations of methyl groups; peaks at 1640, 1620, and 1560 cm^−1^ are attributed to aromatic ring C=C skeletal vibrations; a peak at 1380 cm^−1^ indicates methyl C-H bending vibrations; and peaks at 1250,1189 and 1046 cm^−1^ correspond to O=S=O stretching vibrations [51,52]. Notably, the prominent peak observed at 823 cm^−1^ is indicative of the presence of the C-S bond [53]. These infrared spectral features collectively confirm that the adsorption product is indeed 3,4-dimethylbenzenesulfonic acid. Based on the combined analysis of mass spectrometry and FTIR, it can be concluded that the reaction product of SZO with *o*-xylene is 3,4-dimethylbenzenesulfonic acid. This study not only verifies the high reactivity of sulfonic acid groups in the CB state but also provides critical theoretical and technical support for the application of sulfonic acid-functionalized materials in organic pollutant adsorption.

In this study, anhydrous C_2_H_5_OH was used as an extractant to recover 3,4-dimethylbenzenesulfonic acid from SZO, thereby achieving the sustainable regeneration of the ZO support. Subsequently, the regenerated support was treated with ClSO_3_H in CH_2_Cl_2_ under ambient conditions to prepare SZO (cycles 2~5). Then, this adsorbent was repeatedly used for the reactive adsorption of *o*-xylene. Even after four adsorption and regeneration cycles, the *Q_B_* remained stable at 82.07–78.56 mg/g (Figure S6). This indicates that the regenerated support has good durability and stability. This method not only maintains a high adsorption efficiency but also conforms to the concept of atom economy. A comparative analysis (Figure S7) demonstrates that this method is significantly superior to traditional physical adsorption techniques in the removal of *o*-xylene from gaseous streams [54,55,56,57].

## 3. Materials and Methods

### 3.1. Reagents and Materials

In this experiment, the following reagents and their associated information were utilized: Zirconium dioxide (ZrO_2_, purity ≥ 99.0%) and magnesium oxide (MgO, purity ≥ 98.0%) were supplied by Aladdin Reagent Co., Ltd. (Shanghai, China). Sodium hydroxide (NaOH, purity ≥ 96.0%), potassium hydrogen phthalate (KHC_8_H_4_O_4_, purity ≥ 99.5%), and hydrochloric acid (HCl, mass fraction 36–38%) were obtained from Yongda Chemical Reagent Co., Ltd. (Tianjin, China). Chlorosulfonic acid (ClSO_3_H, purity ≥ 98%) was provided by Aikeda Chemical Reagent Company (Chengdu, China). Furthermore, absolute ethanol (C_2_H_5_OH, purity ≥ 99.7%), methanol (CH_3_OH, purity ≥ 98%), *o*-xylene (C8H10, purity ≥ 99%), and dichloromethane (CH_2_Cl_2_, purity ≥ 98%) were all sourced from Damao Chemical Reagent Co., Ltd. (Tianjin, China). The deionized water employed in the experiment was generated using the laboratory water purification system (model SCSJ-IV, manufactured by Shanghai Hetai Instrument Co., Ltd. (Shanghai, China)).

### 3.2. Synthesis of SMO and SZO

SZO and SMO materials were successfully synthesized via the ClSO_3_H modification method [50]. The detailed experimental procedure is as follows: Initially, 3.0 g of the carrier was precisely weighed and dispersed in 15 mL of dichloromethane solvent. Magnetic stirring was performed at room temperature for 30 min to form a homogeneous suspension (solution A). Subsequently, 2.5 mL of ClSO_3_H was dissolved in 10 mL of CH_2_Cl_2_ to prepare solution B. Solution B was then transferred to a constant-pressure dropping funnel and slowly added dropwise into solution A under continuous stirring. After maintaining the reaction under stirring for 3 h, the solid product was separated by vacuum filtration and washed with 10 mL of CH_2_Cl_2_, followed by another round of vacuum filtration. To ensure the purity of the product, the washing and vacuum filtration steps were repeated twice. The purified product was subsequently dried in a vacuum oven at 60 °C for 12 h. After cooling to room temperature, the product was ground and sieved through a 425-micron (40-mesh) sieve. Finally, the SZO and SMO materials were successfully obtained and stored in a desiccator for further use.

### 3.3. Determination of the Sulfonic Acid Group Loading Quantity

To determine the loading amount of sulfonic acid groups (*Q*_L_, mmol/g) in SZO and SMO, a sample was accurately weighed using an analytical balance (approximately 0.05 g, denoted as *M*). The sample was then placed into a 50 mL conical flask containing 10.00 mL (*V*_a_) of 0.1 mol/L NaOH solution (*C*_a_) at room temperature. A magnetic stirrer was used to mix the solution continuously for 30 min to ensure that all sulfonic acid groups in the sample were fully neutralized. Afterward, phenolphthalein was added as an indicator, and the remaining NaOH was titrated with 0.1 mol/L HCl solution (*C*_b_). The volume of HCl consumed during the titration (*V*_b_) was recorded. Using the experimental data collected, the Q_L_ value can be calculated through Equation (1).

(1) $$Q_{L}=\left(C_{a}V_{a}-C_{b}V_{b}\right)/W$$

In this process, the precise concentration of the NaOH solution (*C*_a_) was determined via calibration with the standard reagent KHC_8_H_4_O_4_. Meanwhile, the concentration of the HCl solution (*C*_b_) was established via back titration using the calibrated NaOH solution. To ensure the reliability of measurements, all titration procedures were performed in triplicate for each sample. Furthermore, for the purpose of comparison, the same calibration procedure was applied to both metal oxide samples (ZO and MO), confirming their negligible acidity (0 mmol/g).

### 3.4. Evaluation of the Dynamic Adsorption Capacity for o-Xylene

To assess the removal efficiency of the SZO material for *o*-xylene in a gaseous environment, this study developed and implemented a dynamic adsorption experimental setup based on a fixed-bed reactor. During the experiment, high-purity N_2_ served as the carrier gas, with its flow rate precisely controlled at 0.050 L/min using a mass flow controller. The simulated gas was prepared via the constant-temperature evaporation of liquid *o*-xylene. Specifically, liquid *o*-xylene was placed in a bubbling bottle, and a low-temperature circulating pump equipped with an ethylene glycol cooling system maintained a constant temperature of 0 °C to ensure a stable inlet *o*-xylene concentration of 7.5 mg/L. The adsorption unit consisted of a quartz tube with an inner diameter of 6 mm and a length of 40 cm, filled with 50 mg of SZO adsorbent and 100 mg of inert quartz sand (particle size range: 0.180–0.425 mm), which improved the uniformity of gas distribution. The residual *o*-xylene concentration in the exhaust gas was monitored in real time using a GC7900 gas chromatograph (Shanghai Tianmei, Shanghai, China), equipped with a flame ionization detector (FID) and a PEG-20M capillary column (30 m × 0.32 mm i.d. × 0.5 μm film thickness). To investigate the effect of the adsorption temperature on the adsorption behavior of the materials, a programmed temperature ramping method was employed, increasing the column temperature from room temperature to 250 °C at a rate of 2 °C/min. Based on the breakthrough curve analysis, the adsorption capacity of SZO material for the target compound under varying temperature conditions was further evaluated. Finally, the removal efficiency of *o*-xylene (*X*_o-x_, %) and the adsorption capacity at the breakthrough point (*Q_B_*, mg/g) were quantitatively determined using Equations (2) and (3), respectively, providing a comprehensive assessment of the material’s adsorption performance.

(2) $$X_{o-x}=\left(C_{\mathrm{in}}\;-\;C_{\mathrm{out},t}\right)\times 100\%/C_{\mathrm{in}}$$

(3) $$Q_{B}=\frac{V_{g}C_{\mathrm{in}}}{m}\int_{0}^{t_{B}}(1\;-\;\frac{C_{\mathrm{out},t}}{C_{\mathrm{in}}})dt$$

(4) $$Q_{m}=\frac{V_{g}C_{\mathrm{in}}}{m}\int_{0}^{t_{m}}(1\;-\;\frac{C_{\mathrm{out},t}}{C_{\mathrm{in}}})dt$$

All adsorption experiments were carried out in parallel with three independent repeated measurements to ensure the reliability and reproducibility of the experimental data.

To characterize the adsorbed *o*-xylene derivatives and investigate the regeneration capability of the SZO material, repeated extraction with anhydrous C_2_H_5_OH was carried out until no fluorescent spots were detected under ultraviolet light during thin-layer chromatography. The solid residue obtained after extraction was rinsed with distilled water and dried at 110 °C to recover ZO. Subsequently, the recovered ZO was treated with ClSO_3_H in CH_2_Cl_2_ solution at room temperature to regenerate SZO. Finally, the extraction solvent was evaporated to yield the desired adsorption product.

### 3.5. Characterization

X-ray diffraction (XRD) patterns were acquired using a Bruker D8 Advance X-ray diffractometer (Bruker, Billerica, MA, USA) equipped with a Cu Kα radiation source (wavelength λ = 0.154 nm). The measurements were performed over the 2θ range of 2° to 90°, with a step size of 0.02° and a scanning rate of 20°/min. Surface textural properties were characterized using a Kubo X1000 (Builder Electronic Technology, Beijing, China) fully automatic specific surface area analyzer. Prior to N_2_ physical adsorption measurements at 77 K, the samples were subjected to vacuum degassing at 120 °C for 2 h. The specific surface area was calculated based on the Brunauer–Emmett–Teller (BET) equation. The Barrett–Joyner–Halenda (BJH) method was utilized to analyze the desorption branch of the nitrogen adsorption–desorption isotherm in order to determine the pore size distribution of the materials. Fourier Transform Infrared (FTIR) spectroscopy was recorded using a Bruker AXS Tensor 27 spectrometer (Bruker, Billerica, MA, USA) within the wavenumber range of 500–4000 cm^−1^. Microstructural features and elemental distributions were examined using a Hitachi SU-8600 (Hitachi, Tokyo, Japan) field emission scanning electron microscope operating at an accelerating voltage of 10 kV. Elemental composition and distribution characteristics of the sample were analyzed by energy dispersive X-ray spectroscopy (EDX) using the Bruker XFlash 6|60 spectrometer (Bruker, Billerica, MA, USA). The test was conducted in the scanning electron microscope (SEM) mode, with an acceleration voltage of 10 kV, a working distance of approximately 15 mm, and a collection time of 60 s to ensure stable signals and reliable data. The elemental types, relative contents, and distribution on the sample surface were obtained through surface scanning, and the data were processed and analyzed using the Bruker Esprit 2.0 software. Thermal stability was assessed using a TA-Q600 thermogravimetric analyzer (Builder Electronic technology, Beijing, China) under a nitrogen atmosphere (flow rate: 50 mL/min). Samples weighing 10–15 mg were heated from room temperature to 400 °C at a rate of 20 °C/min.

Ammonia program temperature desorption (NH_3_-TPD) analysis of SZO and SMO was performed using a PCA-1200 chemical adsorption analyzer (Beijing Prione Electronics Technology Co., Ltd., Beijing, China). The experiments were conducted under a helium purge atmosphere with a heating rate of 10 °C/min. Precisely weighed samples (10.0 mg) were placed in the sample tube and pre-treated at 100 °C under a constant argon flow of 30 mL/min for 1 h to ensure complete dehydration. Subsequently, the samples were cooled to room temperature, and pure NH_3_ gas was introduced at a flow rate of 30 mL/min for 1 h to allow sufficient interaction between the sulfonic acid groups and NH_3_ molecules. Finally, helium gas was continuously supplied at a flow rate of 30 mL/min until the instrument baseline stabilized. Surface chemical compositions were characterized using an ESCALAB Xi+ X-ray photoelectron spectrometer (Thermo Scientific, Waltham, MA, USA). The spectra were calibrated with reference to the C 1s peak at a binding energy of 284.8 eV, employing a pass energy of 50 eV. Reaction products were further identified using liquid chromatography–mass spectrometry (Agilent, Santa Clara, CA, USA, ESI mode).

## 4. Conclusions

In this study, commercially available ZO and MO metal oxides with comparable particle size and surface characteristics were selected as carriers. Two sulfonic acid-functionalized materials, SZO and SMO, were successfully prepared via chlorosulfonic acid modification. The results demonstrated that the sulfonic acid group loading of SZO reached 3.6 mmol/g, slightly higher than that of SMO at 5.5 mmol/g, which was directly attributed to the greater surface hydroxyl density of ZO. The differences in electronic properties between Zr and Mg resulted in the sulfonic acid groups being anchored on the carrier surfaces in the forms of covalent bonds (CB state) and ion pairs (IP state), thereby influencing material performance. Specifically, within the temperature range of 130–150 °C, SZO exhibited a removal efficiency for *o*-xylene ≥ 91.3%, with a breakthrough adsorption capacity ranging from 38.59 to 70.86 mg/g. In contrast, the performance of SMO was relatively weaker. The reaction products were identified as 3,4-dimethylbenzenesulfonic acid through the combined application of HPLC-MS and FTIR techniques. This study provides critical theoretical support for the development of highly efficient materials for the resource recovery and treatment of aromatic VOCs.

## Figures and Tables

**Table 1:** Adsorption parameters of SZO and SMO for *o*-xylene at different temperatures.

Sample	*T*/(°C)	*t*_B_/(min)	*Q*_B_/(mg/g)
SZO	130	17.72	69.64 ± 0.22
	140	20.52	81.18 ± 0.26
	150	9.65	38.17 ± 0.18
SMO	130	0	0
	140	0	0
	150	0	0

## Data Availability

Data are contained within the article. The data presented in this study are available.

## Supplementary Materials

The following supporting information can be downloaded at: https://www.mdpi.com/article/10.3390/molecules30153171/s1, Figure S1. XRD pattern of the samples: (A) SZO; (B) SMO. Figure S2. the NH_3_-TPD curves of SZO and SMO. Figure S3. EDS spectra of the samples: (A) ZO; (B) MO; (C) SZO; (D) SMO. Figure S4. (A) Pore size distribution curves of ZO and SZO; (B) Pore size distribution curves of MO and SMO. Figure S5. X-ray photoelectron spectra (XPS) of the samples: (A) survey apectra of ZO and SZO; (B) survey spectra of MO and SMO; (C) S 2p spectra of ZO and SZO; (D) S 2p spectra of MO and SMO. Figure S6. Reusability of SZO in the repeated adsorption/desorption cycles. Figure S7. Comparison of the adsorption capacity of diverse materials to *o*-xylene. Table S1. Adsorption parameters of SZO for *o*-xylene at different temperatures.
