# Supplementary material for: A Comparative Study on ZrO2- and MgO-Based Sulfonic Acid Materials for the Reactive Adsorption of o-Xylene
Source: Molecules. 2025 Jul 29;30(15):3171. doi: 10.3390/molecules30153171 (PMC12348149; doi:10.3390/molecules30153171)
Supplement: Supplementary file 1 [file molecules-30-03171-s001.zip › molecules-3717644-supplementary.pdf]

Supplementary Information

A Comparative Study on ZrO_2- and MgO-based Sulfonic Acid Materials for the Reactive Adsorption of *O*-Xylene

Hongmei Wang [1], Xiaoxu Zhang [2,*] Ziqi Shen [1], Zichuan Ma [1,*]

1 Hebei Key Laboratory of Inorganic Nano-materilas, College of Chemistry and Material Science, Hebei Normal University, Shijiazhuang 050024, Hebei, PR China; wanghm@stu.hebtu.edu.cn (H.W.); shenziqi@stu.hebtu.edu.cn (Z.S.)

2 School of Environmental Science and Engineering, Hebei University of Science and Technology, Shijiazhuang 050018, Hebei, PR China;

* Correspondence: zhangxiaoxu@hebust.edu.cn (X.Z.);mazc@hebtu.edu.cn (Z.M.); Tel.: +86-0311-80787400 (Z.M.)

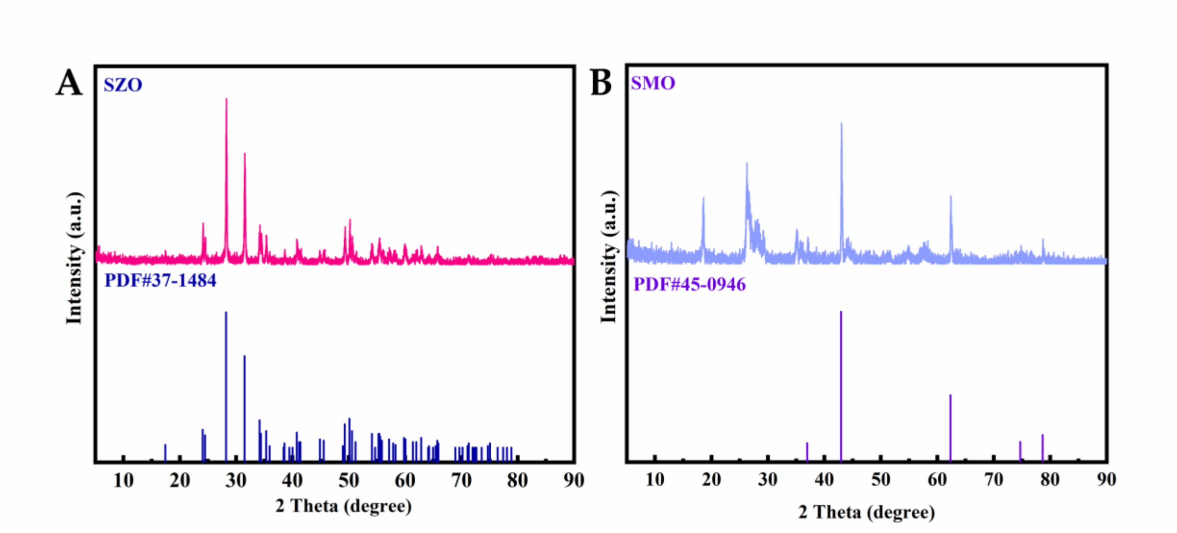

Figure S1. XRD pattern of the samples: (A) SZO; (B) SMO.

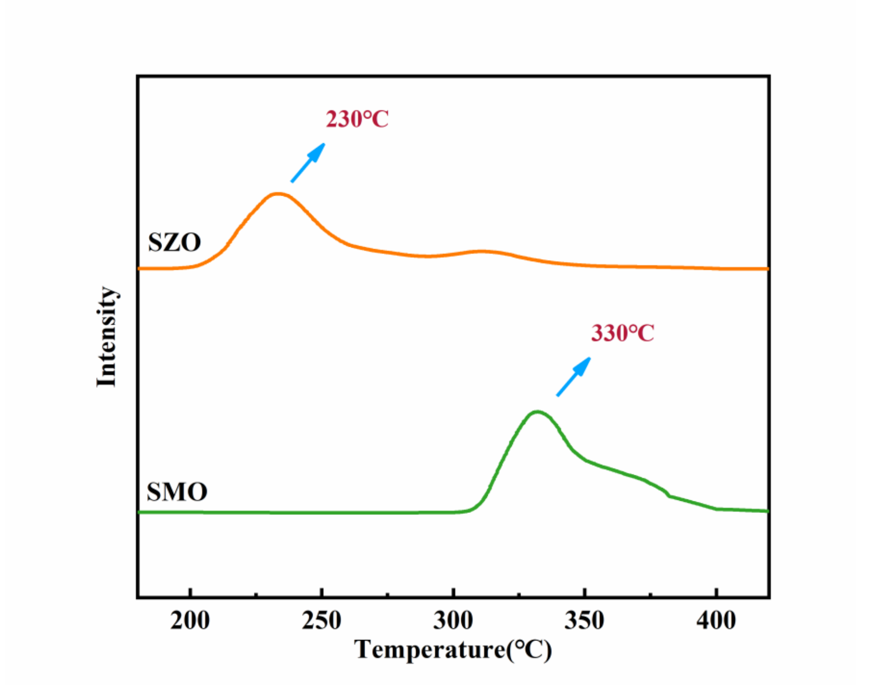

Figure S2. the NH_3-TPD curves of SZO and SMO.

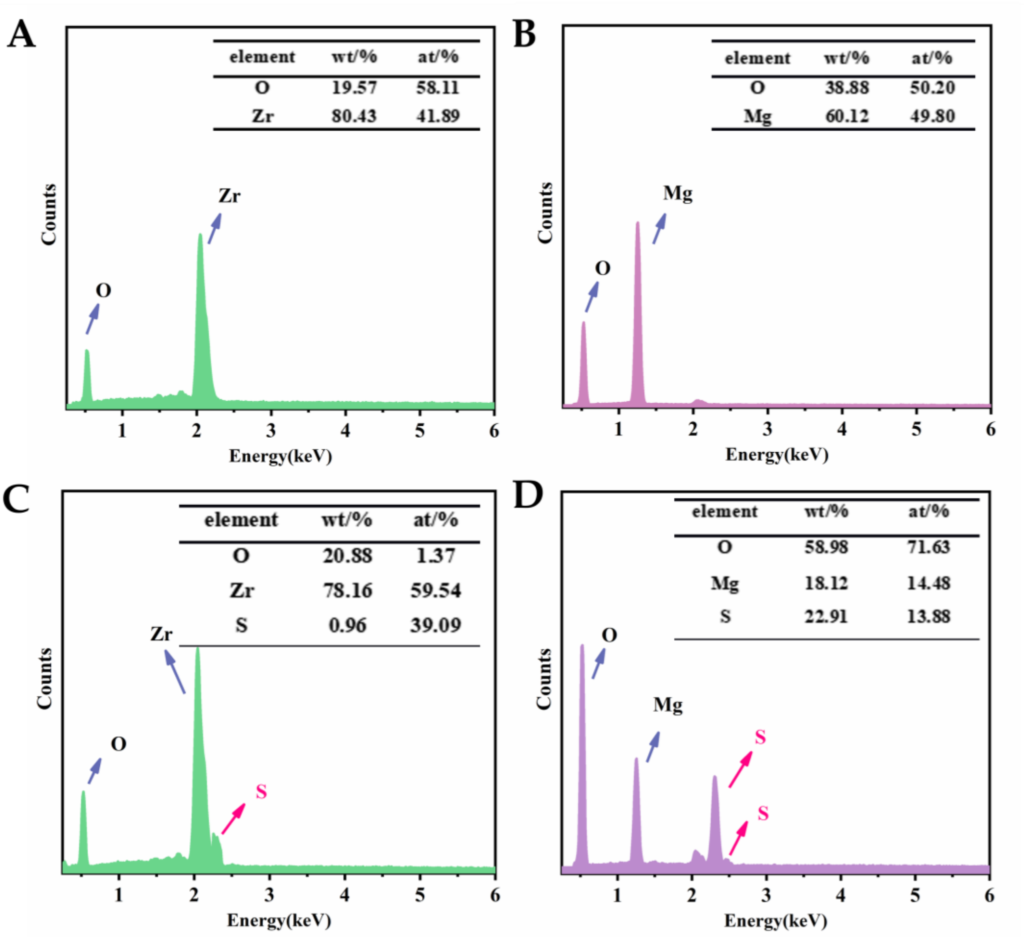

Figure S3. EDS spectra of the samples: (A) ZO; (B) MO; (C) SZO; (D) SMO.

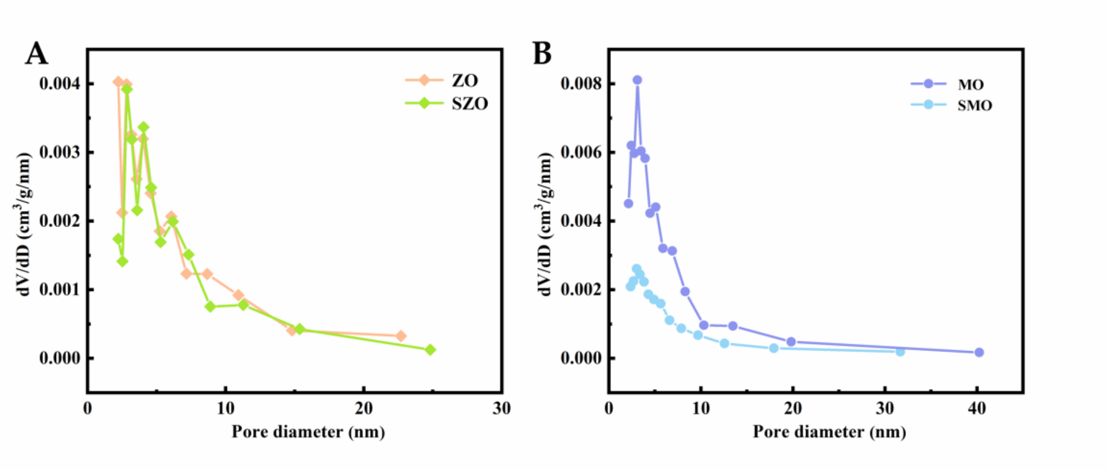

Figure S4. (A) Pore size distribution curves of ZO and SZO; (B) Pore size distribution curves of MO and SMO.

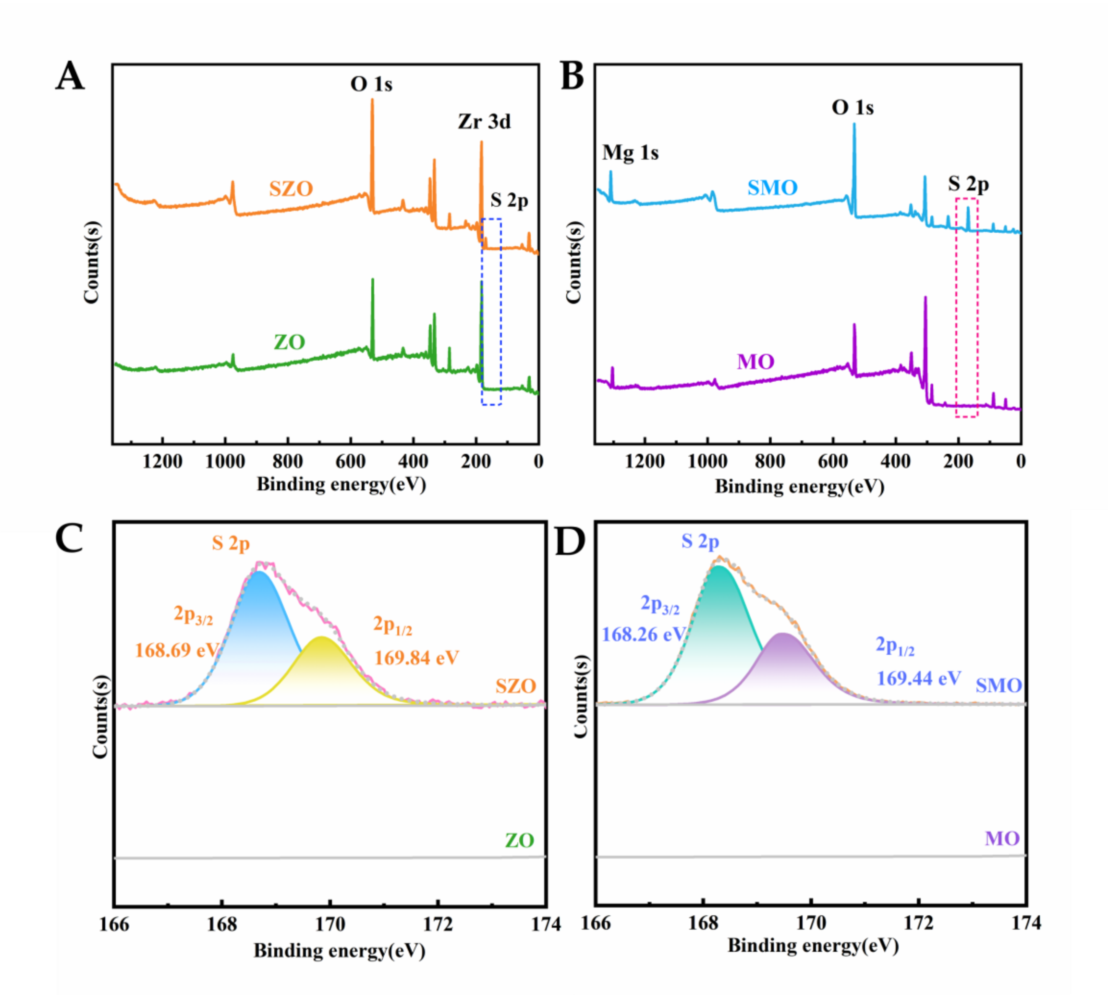

Figure S5. X-ray photoelectron spectra (XPS) of the samples: (A) survey apectra of ZO and SZO; (B) survey spectra of MO and SMO; (C) S 2p spectra of ZO and SZO; (D) S 2p spectra of MO and SMO.

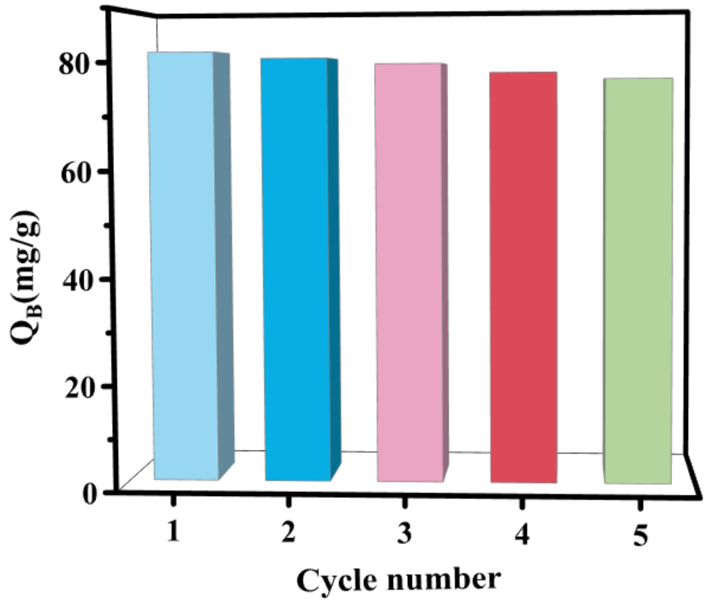

Figure S6. Reusability of SZO in the repeated adsorption/desorption cycles.

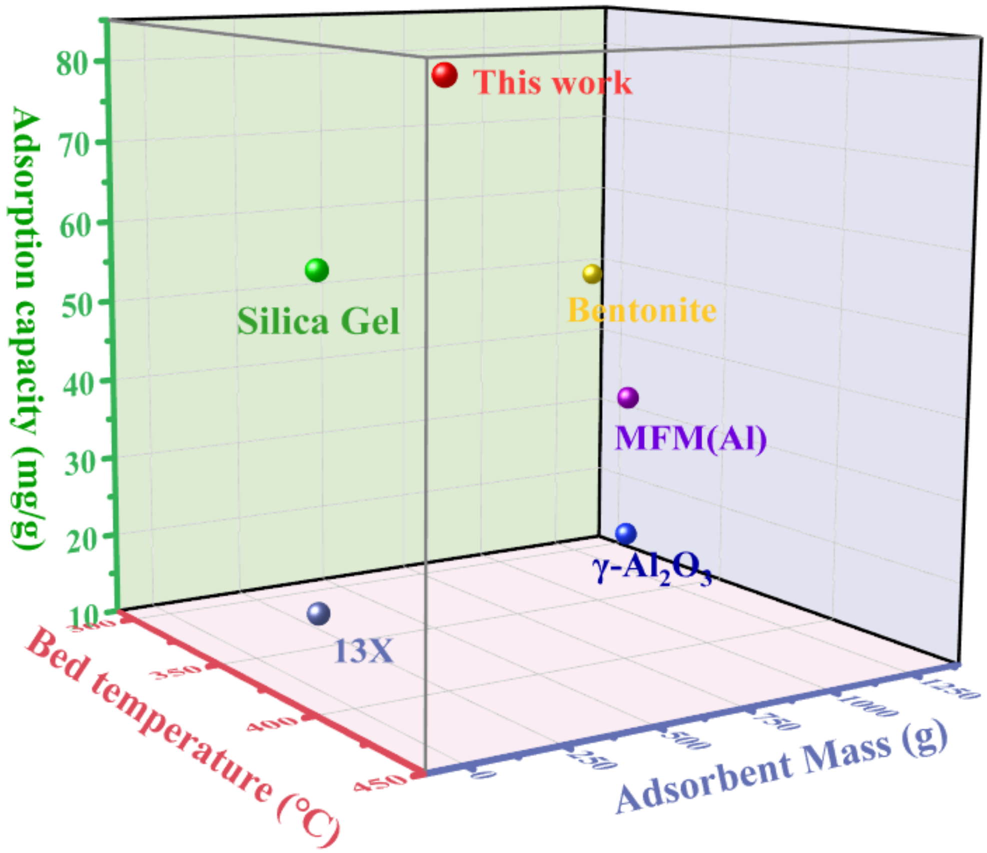

Figure S7. Comparison of the adsorption capacity of diverse materials to *o*-xylene.

Sample	T/(°C)	t_m/(min)	Q_m/(mg/g)
SZO	130	50.61	109.43 ± 1.46
	140	53.65	87.15 ± 0.38
	150	51.13	99.76 ± 0.54

Table S1. Adsorption parameters of SZO for *o*-xylene at different temperatures.
